# On Paralysis and Diseases of the Brain
*Clinical Lectures on Paralysis, Disease of the Brain, and other Affections of the Nervous System. By Robert Bentley Todd, M.D., F.R.S., Physician to King's College. 8vo. London: John Churchill, Princes-street, Soho. 1854.


**Published:** 1854-07-01

**Authors:** 


### Art. II.—ON PARALYSIS AND DISEASES OF THE BRAIN.*

We have always considered the record of clinical observations as the best mode of imparting practical instruction, and we wish it was more frequently adopted, in lieu of the elaborate treatise. It is true that the clinical lecture is open to the charge of *tautology;* but even the repetition of important points may strengthen rather than weaken the interest of the subject. Remarks and notes made at the bed-side are indeed specimens of the natural history of disease freshly gathered, and not a mere *hortus siccus* of pathology, concocted from the stores of a well-stocked library. In the faculty of elucidating the salient points of a case, and forming deductions therefrom, we know few superior to the author of this work. His *truthfulness* is not, perhaps, the least merit of Dr. Todd,—he acknowledges having occasionally fallen into premature conclusions. This is the track of true philosophy, and if any one can *afford* to confess occasional fallibility in our most intricate and multiform science, it is this eminent physician. Indeed, no one knows better than himself the value of these confessions

* Clinical Lectures on Paralysis, Disease of the Brain, and other Affections of the Nervous System. By Robert Bentley Todd, M.D., F.R.S., Physician to King's College. 8vo. London: John Churchill, Princes-street, Soho. 1854.

to the student, feeling that we often learn more from the *negative* points (so to write) of pathology than from the proudest recital of our successes. In the words of the author:—"The successful cases speak for themselves, the failures we would fain throw a veil over; but, be assured, in so doing we benefit neither science nor ourselves."

Dr. Todd in the early part of his volume refers to the distinction between the paralysis of *sensation* and *motion*, the former being the most amenable to remedial treatment, and then observes that paralysis is not to be deemed a *disease*, but a *symptom;* the secondary, or even ternary result of remote disease. The author refers at length to the causes of paralysis,—those conditions which interfere with the propagation or transmission of nerve influence or force, a morbid state of the nervous centres or their trunks, blood poisons, inflammation, atrophy, hardening or softening, hyperæmia, anæmia, concussion, compression, or division of nerve. The pathological anatomy of the centres of volition is so important a preface to these discussions, that we will quote the author's remarks on this intricate point:—

"What are the causes which may give rise to paralysis? These are, either an affection of the nerve or nerves, whose power is destroyed, in some part of their course, or a morbid state of the centre in which the nerve or nerves are implanted, or with which they may be less directly connected. The nervous trunks themselves may be impaired in their nutrition, the centre being healthy, or they may have suffered some mechanical injury from violence or pressure; thus either they become imperfect conductors of the nervous force, or they are rendered altogether incapable of propagating it; or some portion of the centre of volition is the seat of a morbid process, whereby the influence of the will over certain parts is suspended, and thus the nerves of those parts receive no impulse at all from that centre, whether mental or physical; and although perfectly healthy in themselves, are incapable of taking part in voluntary acts.

"Whatever interferes materially with the conducting-power of nerve-fibre, or the generating power of nerve-vesicles (grey matter), will constitute a paralyzing lesion.

"I would say that the centre of volition is of very great extent: it reaches from the corpora striata in the brain down the entire length of the anterior horns of the grey matter of the spinal cord, and includes the locus niger in the crus cerebri, and much of the vesicular matter of the mesocephale and of the medulla oblongata. Disease of any part of this centre is capable of producing paralysis; but as the intra-cranial portion of it exercises the greatest and most extended influence in the production of voluntary movements, so disease of this portion gives rise to the most extended and complete paralysis.

"Another fact which I would impress upon you is one which anatomy in a great degree demonstrates, and which pathological research confirms—that the centre of volition for either side of the body is not altogether on the same side of the body. Of the centre for the left side of the body, for instance, the intra-cranial portion is on the right side, and the intra-spinal portion on the left side, and these two portions are brought into connexion with each other through certain oblique fibres from the anterior pyramidal columns of the medulla oblongata, which cross from right to left, decussating with similar fibres proceeding from left to right, which belong to the centre of volition for the right side of the body."

Of the paralysis from lead-poison, the prominent signs are hand-drop, atrophy of limb, and those curious blue lines on the margin of the gums. Dr. Todd believes in a peripheral affection travelling from the surface to the centre, and *then* we have " epileptic convulsions and other symptoms of centric disease." Lead has been detected in the brains of these patients. The treatment advised for these cases consists in the elimination of the poison by various depurating excretions, the sulphur bath, galvanism, iodide of potassium, citrate of iron, and change of air. The cases of paralysis from injury to nerve tissue seldom admit of perfect recovery, owing to the imperfect regeneration of such tissue.

In illustration, we refer to a passage in page 75 of the book:—

" When the paralysis has been caused by mechanical injury, your prognosis must generally be unfavourable, more especially if any distinct solution of continuity have taken place in the nerve. Nerve-substance is very slow of regeneration; and when it is reproduced, the new fibres do not adapt themselves with precision to the old ones, and so they form very imperfect conductors of the nervous force."

Hysterical paralysis is an obscure malady, as, indeed, are all *local* hysterical affections; for instance, the three cases described by Sir Benjamin Brodie. Dr. Todd has, however, referred in the following passage to a diagnostic sign:—

" If you look at a person labouring under ordinary hemiplegia from some organic lesion of the brain, you will perceive that, in walking, he uses a particular gait to bring forward the palsied leg: he first throws the trunk to the opposite side, and rests its entire weight on the sound limb; and then, by an action of circumduction, he throws forward the paralyzed leg, making the foot describe an arc of a circle. Our patient, however, does not walk in this way; she drags the palsied limb after her, as if it were a piece of inanimate matter, and uses no act of circumduction, nor effort of any kind to lift it from the ground; the foot sweeps the ground as she walks. This I believe to be characteristic of the hysterical form of paralysis."

In the treatment, which is of course that of general hysteria, *we* have proved the efficacy of the valerianate of zinc.

The *one-sided* indications of paralysis from cerebral lesion must be distinguished from those of spinal causation. These are of course *centrifugal*, and are generally occurring in persons of intemperate habit. The local pains in the head often resemble the sensation from a nail driven into the head,—*clavus hystericus.*

The indications of *irregular* muscular action are often striking,—a derangement of the normal antagonism of action, as illustrated by double vision, &c. The *pain* from disease of the membranes is of course usually more severe than from that of the medulla,—as pleuritic exceeds pneumonic suffering.

The most common seat of lesion is the lateral ventricle, the corpus striatum and optic thalamus: blood, pus, or water being often discovered as the result of morbid action.

Of the four conditions of the muscles in paralytic limbs, Dr. Todd offers some accurate remarks. They are too long for quotation, but demand the close attention of the morbid anatomist.

The author differs somewhat from Dr. Marshall Hall, regarding the *hyper-excitability* of a paralyzed limb. He believes, however, that in *spastic* rigidity they may be so, but that in complete muscular atony they are *far less* excitable than those of the sound side.

The diagnosis between mere facial and cerebral affections is of the highest importance, as the decision on the first form may enable us to pacify the anxiety of patients and friends as to the result. We quote the following passage on this point:—

"It is remarkable how seldom the seventh pair of nerves is affected by disease of the brain. I cannot say that I ever saw an instance of complete paralysis of the orbicular muscle of the eyelids due distinctly to uncomplicated disease of the brain; and I have only seen a few in which the power of the muscle appeared to be enfeebled from that cause. Thus we have a point favourable and consolatory to a patient afflicted with *portio dura* paralysis; namely, that the affection being seated in that nerve need not excite the same alarm as to disease of the brain as in other cases of partial palsy, that of the third nerve, for instance. Moreover, disease of the brain would give rise to a different form of facial palsy."

On the subject of the upward movement of the eyeball, the author speaks in controversion of Sir Charles Bell's theory, although on the subject of facial neurology generally he eulogises the acuteness of that learned physiologist.

"If" (writes our author) "you will take the pains to watch persons sleeping, whenever you have the opportunity, you will find that in *sound* and *tranquil* sleep there is no indication of active contraction of the orbicular muscle: there are no wrinkles of the eyelid, and no depression of the brow, as when that muscle is in strong contraction;—if, with the greatest care and gentleness, you raise the upper lid, you will find the eyeball directed forwards, maintained in this position by the equilibrium of its muscles. Should your attempt to raise the lid give rise to a reflex action, you will encounter a distinct resistance from the contraction of the orbicular muscle, and the eyeball will be turned upwards and inwards, more or less forcibly in proportion to the force of the reflex action."

Too much stress cannot be laid on the subject of otitis and affections of the petrosal structure in reference to paralysis. These deeper maladies must be distinguished from the primary or *peripheral* affections (of which they may, however, be sometimes the result), which occur in certain subjects; the rheumatic, for instance, from a mere current of cold air. We have been long convinced of the fact described in the following quotation:—

"Periodical neuralgic affections are, I believe, very frequently due to the determination of some poison to a particular nerve—as the paludal poison, or some matter generated in the system, gouty or rheumatic. There is no reason why such morbid matters should not affect a motor nerve as they affect a sensitive nerve, causing paralysis in the one case, and neuralgia in the other."

We might be inclined, perhaps, if hypercritical, to object to the term *rheumatic paralysis*, as there is rather a *reluctance* than an inability to muscular action. The fear of pain suppresses it; a hero would move a limb thus affected; a coward even might run from danger; but in true paralysis, however strong the will, the power is lost. The recorded cases, associated with rheumatism and gout, are very *apropos*.

In these cases, the remedy recommended is iodide of potassium with galvanism. In many cases we might prefer the more specific colchicum, as we would combine quinine in intermittent maladies.

The author objects to the use of blisters, as they may irritate the cervical glands. We have not seen such effect produced by the fluid preparations of lyttæ.

We think there might be more discrimination between spasm and paralysis. The want of antagonism may be a clonic spasm on the same side as the lesion, and we may thus avoid a long discussion on the *cross*-pathology from interlacing of fibres, &c.

In cases of cerebral lesion, it is very difficult often to decide on the exact locality. Clots in various tissues may sometimes be followed by similar symptoms. Perhaps the *hydraulic compensation* within the cranium may somewhat explain this dilemma. A clot may be found in a yielding or soft medulla, but the compressing effect may be only *felt* where a firmer structure first offers resistance.

The cases of diseased brain recorded in the sixth lecture strikingly demonstrate the two causes or conditions, the predisposing and exciting. Where a certain diathesis—as of gout—exists, and cerebral participation is indicated, we should enjoin great caution, as a very slight excitement may light up a local action not to be controlled. Thus Dr. Todd refers to the state of white ramollissement, the capillaries also becoming diseased, in which a slight exertion causes them to yield, and a clot at once ensues.

These cases are often combined with a protracted stage of renal disease, which the author terms "gouty kidney," and with atheromatous deposits on the arterial tissue. The tracing of morbid progress in these maladies (p. 114) is very scientific, and proves how very slight causes may induce obstructed circulation, and eventually concentric hypertrophy, which is indeed the *ruse* adopted by nature to overcome obstruction by the addition of muscular force; so intricate are Nature's modes, and consequently the study of our profession.

This lecture is concluded with some remarks adverse to the *routine* treatment of apoplexy. We quote this conclusion as highly practical:—

"If, upon full inquiry into all the particulars of the case, you find that your patient is of full plethoric habit, with too much blood in his body, and with a sufficiently strong heart, you may bleed him with every chance of success; but if he has been of intemperate habits, is labouring under organic disease of the heart and arteries, is of gouty or rheumatic constitution, then, whatever popular or medical custom may say, my advice to you is, hesitate much before you deplete by bleeding.

"The objects which it is proposed to gain by bleeding are a diminution of the cerebral congestion, and the stoppage of the hæmorrhage into the brain; and where it is quite clear that cerebral congestion does exist, and that that congestion causes the cerebral hæmorrhage, this is clearly a rational practice. But you must bear in mind that in a large number of the cases—probably the majority—there is in reality no cerebral congestion, and that the hæmorrhage is of a kind not likely to be stopped by taking away blood—by establishing another hæmorrhage elsewhere.

"On the whole, then, I think that the results of experience denote that the majority of cases of apoplexy are best treated by purging, shaving the head and keeping it cool—perhaps blistering, and that bleeding is rarely applicable, except to the young, vigorous, strong, and plethoric."

In the seventh lecture there are some valuable remarks on diagnosis. This chapter contains a special reference to *arcus senilis*, as a symptom of cerebral disease.

The more we see of encephalic disease, the more are we conscious of its obscurity, and of the importance of tracing these affections to their right causes. How often have symptoms been referred to disease of nerve tissue, when they have originated from poisoned blood?

In cases of coma, especially occurring in the débauché, we must not look alone to the *head.* It is most essential that we ascertain the state of the renal secretion. If this be very scanty, or nearly suppressed, there can be little doubt of the presence of urea in the blood circulating in the brain. Even if the urine be in large quantity, it may yet poison the brain blood if it be of low specific gravity, and fails in eliminating the *solids* of the urine. In an analysis by Dr. Beale, the urine of a patient who passed five pints daily "contained twenty-two parts of solid matters in a thousand, and these consisted chiefly of *albumen and extractive matters*, whereas twelve or fourteen parts, at least, ought to have been urea." In such a case, coma and convulsions are very likely to ensue. In illustration of these facts, we quote (by anticipation) the following test from the sixteenth lecture:—

"A blister was applied to the back of the neck; and when it rose, the serum was carefully collected, and tested for urea. The whole quantity of serum was evaporated to dryness over a water-bath, and the residue was extracted with alcohol, which is a ready solvent of urea. This alcoholic extract was then evaporated to dryness, and a little water added so as to make a syrupy mass, which was plunged into a freezing mixture, and a few drops of pure nitric acid were added. If urea be present, the characteristic crystals of nitrate of urea are soon formed in the solution, and may be recognised either by the naked eye or by the microscope."

In this *renal epileptic coma* Dr. Todd employs blisters, purgatives, and diaphoretics. The best purgative being elaterium, of course cautiously administered.

In the cases of the delirium of drunkards we have often witnessed the injurious effect of agitation and exertion; for they are often as sensitive as the *mimosa.* The author is of the same opinion:—

"About two years ago a man was admitted here for epileptic delirium. Finding that his delirium was very noisy, and disturbed the other patients, I had him placed in a separate ward, where he recovered from his delirium. It was found necessary to move him upstairs, and shortly afterwards he became delirious again, and died comatose.

"I am satisfied, from these and other cases, that there is nothing respecting which we ought to be more cautious than as to moving patients either in or just recovered from delirium; even to move them from one room to another on the same floor is dangerous, still more moving to any distance, or to another floor. Let us take this case as a warning of the necessity of great caution and circumspection before we sanction the removal of a patient under such circumstances."

The subject of Hemiplegia occupies seven lectures, and we deem it the most valuable monograph we possess on that affection.

Dr. Todd remarks that the *reflex actions* induced by peripheral excitement are attended by great uneasiness from "an irritable state of the sentient nerves and of the centre of sensation."

He adds:—

"There is, however, a curious and very interesting involuntary movement, which you will sometimes witness in hemiplegic cases. It occurs simultaneously with yawning, and less frequently with the actions consequent on emotion, surprise, joy or pleasure, or grief, as in laughter or crying. I may here mention that yawning is a very frequent, and sometimes a troublesome, and not always a favourable symptom after an attack of hemiplegia. It is more frequent in proportion as the shock is severe, but it seems to come on, as the first effects of the shock are declining."

We have known practitioners apply friction to the *drawn side;* we were, therefore, not surprised to read:—

"'His face,' the patient's friends will tell you, 'is all drawn on one side;' and they will hardly believe you when you assure them that the drawn side is all sound, and that its being drawn is merely the result of the want of a resisting power on the opposite side."

The mechanism of cerebro-spinal action is of the deepest interest; in its consideration we should have the distribution of the nerves ever in our mind's eye. Of the *rationale* of hemiplegic palsy we read as follows:—

"You know that paralysis may be caused by any lesion which interrupts the continuity of a nerve or set of nerves, and which interferes with the due connexion between these nerves and the centre of volition; or by lesion of the centre of volition itself. Thus, then, you may have hemiplegia dependent on peripheral affection of the nerves, the morbid process spreading from periphery to centre—this is a rare and an incomplete form of hemiplegia,—or you may have it caused by a lesion in some part of the brain or spinal cord. If the lesion be situated within the cranium, above the point of decussation of the pyramidal columns of the medulla oblongata, the palsy will be on the side of the body opposite to the lesion: this is the most common form of hemiplegia. If it be seated in the spinal cord, below the decussation, the palsy will be on the same side of the body as the lesion; but in such a case, which is very rare, the phenomena present certain very essential points of difference from cerebral hemiplegia."

Then follows a description of these six forms, into which Dr. Todd divides hemiplegic affections,—lesion of the brain—that of the spinal medulla—epileptic hemiplegia—choreic hemiplegia—hysterical hemiplegia—and peripheral hemiplegia. In comparing the surface sensibility, Dr. Todd employs the compasses method of Weber, on approximating the points of which on the paralyzed side they seem to the patient as one—on the sound side, as two. After deciding that ramollissement results from diseased or plugged arteries, Dr. Todd divides the cases of cerebral hemiplegia into three classes, depending chiefly on relaxation and rigidity of muscle; and it is clear that, in regard to treatment, this division is of vital importance. Atonic hemiplegia may occur in two modes, with or without the state of coma. The whole of the author's remarks cannot be read too carefully. We have only space to quote the passages referring to the proximate cause:—

"The evidence now accumulated respecting the lesions which give rise to these two forms of hemiplegia, indicate, I think, very distinctly that they result from defective circulation through the brain, and enfeebled nutrition of the cerebral matter. In some instances actual obstruction of important arterial channels can be shown; in others, there is a marked degeneracy of a large portion of the arterial and capillary system which may have preceded or gone on simultaneously with the cerebral degeneration. In all cases the cerebral disease reaches such an extent, that the vesicular matter imperfectly generates the nervous force, and the fibrous matter becomes a bad conductor of it, or even a non-conductor, or its continuity is interrupted, and so its power of conduction is rendered mechanically impossible. And, if the softening of brain have been of sufficient duration, there will be found in it the large vesicular bodies, containing fatty particles in a state of minute division, which indicate a further degeneracy of the brain tissue, or an attempt at a reparative process."

The treatment consists in semi-recumbent repose, enemata, in cases of obstinate constipation, large doses of calomel or croton-oil; ammonia or chloric ether subsequently.

The following suggestions close the lecture:—

"It sometimes happens that in these cases a rigidity of the muscles comes on very early, which indicates an inflammatory process going on around the clot, which may end in the formation of pus and abscess, and is to be combated by the use of mercury. But you must be careful to distinguish this from the muscular rigidity which is of late occurrence, and results from a restorative effort of nature; and with which it is therefore not desirable that you should interfere."

The cases of hemiplegia with *rigid* muscles are divided into those of *early* and *late* rigidity. The author's idea of the cause of slight and partial forms is:—

"That it depends upon a state of irritation, propagated from torn brain to the point of implantation of the nerves of the affected muscles. But, you will ask, why is it that in some cases of clot the hemiplegia will be accompanied with complete relaxation of muscles, while in other cases the rigidity of which I have spoken exists? The answer to this question is as follows: in the cases where there is no rigidity, the clot lies in the midst of softened brain, and has not in any degree encroached upon sound brain; but when rigidity exists, the clot has extended beyond the bounds of the white softening, and has torn up to a greater or less extent sound brain."

It seems that both irritation—*i. e.*, a state of exalted polarity, or high tension of nerve tissue—and inflammation of brain may induce this rigid palsy. Both may interfere with the conducting force of nerve fibre, or the productive faculty of vesicular tissue. "A paralyzing lesion is also perfectly compatible with an irritative one."

The second form of late rigidity is sometimes tetanic, and is often associated with inflammation. Dr. Todd considers this a most perilous form—many dying early after the onset. In some the rigidity lapses eventually into relaxation, the muscles then becoming atrophied in an extreme degree. In one case, under the author's care, the disease, resulting from inflammatory ramollissement, the deltoid and the scapular muscles became so attenuated that the head of the humerus actually fell out of the glenoid cavity. There are few protracted cases of this kind that are not marked by rigid digital flexion both of the hands and feet; often that of the popliteal and calf muscles. The author accounts for the causation of this state by a shrinking of cerebral tissue consequent on the effort at cicatrization, the muscles becoming hence both irritated and atrophied. In one hemiplegic patient, a lady of sixty, a very curious psychical phenomenon was observed—the substitution of one word or name for another. On her recovery even, she always misnamed the members of her family.

*Peripheral hemiplegia*, the "creeping palsy" of Cheyne, is so named by the author from "the mode of access of the paralysis;" the first sensation being that of numbness of an extremity, followed by progressive diminution of power, and then of temperature.

Hysterical hemiplegia is a condition to which our thoughts have been long directed. But how can we define it, seeing that we know so little of that we term hysteria? It depends probably on some mystic fault of innervation which, with all our microscopes, will probably ever elude our search. That form of enervation we term aphonia often comes and goes instantaneously—it is a sort of hysterical Jack in the box. In former numbers of this Journal we have referred especially to two interesting cases of this form. The one was a married lady, whose paroxysms always came on about midnight: the cause was evidently emotional. The subject of the other was a very beautiful girl of fifteen, in whom aphonia existed for many months, the only moment of articulation being the pronouncing the name of a jewel in the exhibition, which was to be her own if she named it. Among many interesting cases related by Dr. Todd is that of a male hypochondriac who became completely aphonic from extreme excitement.

Of the forms of epileptic hemiplegia very interesting cases are related by the author. The remedies recommended are valerianate of zinc, sumbul, cod-liver oil, iodide of potassium and steel, purgatives, and the shower-bath.

With Dr. Todd's anathema of the congestive hypothesis we do not quite coincide. He asks—"What is the paralyzing cause, when the paralysis is so transient as to pass off in a few minutes?" We confess we have been wont to explain this by reference to the forces or conditions of the blood, however the author may term it a clumsy explanation, and that the vessels are only secondary elements in the construction of the organ. We believe that, however, the "elements of the tissue" may *predispose,* any exciting cause in the lungs or the heart, and especially if that be attenuated, may instantly induce the congestive state, and the disorder itself of which we are writing. Nay, without the "morbidly excited polarity," as it is termed, of nerve-tissue, hyperæmia, excess or poisoning of blood, may be sufficient for the explanation, however we may conclude that ramollissement and clot may form the gist of our arguments in the severe and fatal maladies.

The observations of Dr. Todd on the anæmic condition of the brain in some cases of coma are most judicious. He very truly observes, that the erect posture induces the attack, and the recumbent relieves it. The fact seems to point to the acceleration of pulse in the erect position as analogous to the hæmorrhagic effort. The fashion of routine depletion in sudden seizures has been long on the wane. We believe there can be but few very old women of the very old school who would now call for their *porringer* and draw out their lance, when they are summoned to a *fit.* Some of these cases, indeed, can only be saved by stimulants; and the author cites an interesting case from Mr. Stokes, in which the withholding of stimuli and the blistering of the head induced prostrate collapse, from which a renewal of stimuli completely recovered the patient, who subsequently, however, died from mitral disease. On the proximate cause of this form, thus writes our author:—

"Chorea being due to a disturbed nutrition of some part of the brain in intimate connexion with the centre of volition, the disturbing cause may act exclusively on one side of the brain, or it may operate more on one side than the oeher. The effect of this disturbance is first manifested in an irritative state, creating the choreic movements, and this passes sooner or later into an exhausted or paralytic state."

With Dr. Todd we believe that this proximate cause will be much elucidated by subsequent examination of the tissues constituting, as we believe, the centres of volition and emotion. Regarding the specific gravity of cerebral tissues considerable difference has been observed. Dr. Aitken found in a choreic subject that the corpus striatum and optic thalamus on one side were 1·025, on the other 1·081. Dr. Todd believes that syphilitic contamination is often at the root of the epileptic attack.

The principles of treatment in these cases seem to be the restoration of systemic health, and the employment of passive motion. Depletion, if absolutely required, should be sparingly employed, and only at the onset of attack. Mercury should be administered only in cases of cerebitis or syphilis, and altogether rejected when albuminuria exists. The proximate causes of hemiplegia are never of course to be sought for much below the decussation of the anterior pyramids. One very interesting fatal case is recorded, in which the spinal cord on the left of the median fissure was compressed and flattened by an enlarged odontoid process.

The influence of the poison of lead is, in the opinion of the author, peripheral, *i.e.*, the muscles themselves are primarily affected, then, if protracted, the nerve tissue, and subsequently the brain.

With his usual zeal in search of truth, Dr. Todd has submitted different portions of the brain to a very scientific examination, both to compare its specific gravity and detect lead poison, and also for the discovery of the tritrate of urea in cases combined with evident renal disease. The process was very successfully conducted by Mr. Conway Evans. Dr. Todd adds in a note:—"I have sought in vain for evidence of the presence of carbonate of ammonia in the expired air and in the blood, as suggested by Frerichs."

When syphilis affects the dura mater its state is probably analogous to that of the periosteum, and we have an interesting examination of the brain of a patient dying in this state. The dura mater, extremely thickened, adhered to the right parietal bone, and also to the visceral layer of the arachnoid, the cerebral tissue being partially red and softened. In cases of this description, Dr. Todd strongly recommends the iodide of potassium as a specific in removing the morbid deposit; but it is requisite to follow up this by other remedies. The author's judicious precepts on this point deserve quotation:—

"In such cases we must trust to the repeated use of iodine as one element of cure, care being taken to watch the constitution of the patient during its administration. And we may aid the influence of the iodine, by the occasional use of mercury, either at the same time with the mercury, or, as I prefer it, alternately,—that is, giving first a short course of mercury, then of iodine, then of mercury, and then omitting both, and using only tonic means, both medicinal and hygienic, resuming, if occasion should demand, the mercurial and iodine treatment. And you will also find great benefit from the prolonged use of well-made decoction of sarsaparilla, or of cod's liver oil, or of both."

In the relation of the very interesting and rare case of idiopathic trismus, Dr. Todd alludes to the influence of salivation in inducing that state.

The pathology of chorea is quite as obscure as that of hysteria, of which indeed (and we must adopt the term hysteria for want of a better) it is but a variety, occurring in subjects of the same diathesis, and constantly excited by some emotional influence. As the attack is usually sudden, often in consequence of a shock, a sort of *similia similibus* mode seems to be the kind of remedy most efficacious—such as the douche, or splashing, or ablution with cold water. Quinine, or the liquor cinchonæ, iron, and the most nutritive diet should be added. In cases of attenuation, cod-liver oil is very useful. If the urine possess high specific gravity, the additional remedy is obvious. If ascarides exist, enemata of salt and water are useful. The concluding lecture of Dr. Todd, however, illustrates the most important form of local hysteria, on the correct diagnosis of which not only the reputation of a physician but life itself may depend. There are few who have not occasionally been placed in dilemma by cases which called for the closest and most patient attention ere the mind could be satisfied of their nature. The case of Harriett B——, related by the author, is of this kind, and as we have seen and have recorded several analogous cases in our Journal, we point to them as far more psychical than organic. As a proof of the phenomenon of psychopatheia, as a late author has termed it, Dr. Todd thus writes :—" There is another very important feature in the case which deserves especial notice—when her attention is much engaged she certainly suffers less." We have been aware of conditions of hyperæsthesia of the skin, in which if even a feather were dropped on the abdomen, the patient shrieked with agony, and yet if the attention could be intensely fixed on another subject, pressure could be borne without suffering. It is clear this unconsciousness could not be were inflammation present. It is evident that an erroneous or morbid notion of self lies at the root of this puzzling malady, which may assimilate indeed the most acute form of peritonitis if pain only were regarded in the symptomatology. Dr. Todd reasons very scientifically on his case, alluding, in the formation of his diagnosis, to other signs, as the prominence of the upper lip, the languid drooping of the eyelids, leucorrhœa, amenorrhœa, &c., which, if duly studied, will lead even the young student away from error, and prompt him to employ antispasmodics instead of depletion ad deliquium, perchance ad mortem.

The irritable uterus and breast offer also very prominent examples of this local hysteria. They are all, however, so analogous in character, though variously localized, that the history of one may elucidate the entire class. Whenever, therefore, we are consulted in a malady (especially if it be of a young female) the name of which we finish by *dynia* or *algia*, we cannot be too careful in our decision as to whether the blood or the nerve be the seat of the proximate cause.

In his allusions to gastrodynia, for instance, Dr. Todd remarks :— "We must be very careful to distinguish this from the pain resulting from ulcer of the stomach," &c. Perhaps the diagnosis here is more clear than usual, as injesta invariably aggravate the inflamed or ulcerated membrane, whereas irritable gastrodynia is as constantly relieved by them.

We feel half disposed to follow up this most interesting and important subject, as it is precisely in the class of persons whose cases we are now discussing in which this softened and abraded mucous membrane imperceptibly extends and deepens to the other coats of the stomach, until after a full meal, or some extraordinary exertion, the tissues yield in a moment, and the gastric contents ooze out into the peritoneal cavity; and this without any especial attention to the case, or unfavourable prognosis. These cases are more common than many believe. We have seen constantly exhibited to the Medical Society of London specimens of this perforation of the stomach, in which the orifice looks as if *punched out* by an instrument. There is a most interesting case recorded in the last volume of the Society's Transactions.

We may observe *en passant* that in nervous gastrodynia we have experienced the greatest benefit from the subnitrate of bismuth and henbane, together with frictions of camphorated spirit and wine of opium to the region of the stomach.

"Hysterical spine," as the author terms it, comes under the same category; the pain is extensive, often along the whole column, and hyperacute, thus distinguishing it from caries, which is less severe and limited to a spot. In these forms of hysteria, therapeutics seldom fulfil our wishes; psychical remedies are often more gratifying; change of scene and air, and pleasurable concentration of the thought away from the painful spot, will seldom fail in relieving if they do not entirely remove the malady. Cataleptic affections sometimes form a part of hysteria, the muscular fibres being unusually rigid. The psychical phenomena are in these cases rather anæsthetic as to external agency; to a slight blow or pinching the patient is often totally insensible although perfect consciousness to all other points exists. On the obscure nature of hysteria we did not expect that even so acute a pathologist as Dr. Todd could enlighten us much; original constitution of nerve tissue, defective assimilation, unhealthy blood, are the chief physical causes, according to our author, and they lead us of course to amend these faults by the nutritive and tonic plan. In the relief of the paroxysm, cold water splashing, camphor mixture, and ammonia, and hyoscyamus, with enemata of turpentine, and assafœtida are the chief modes adopted by the author.

It will be perceived, from the very slight points of difference between the learned lecturer and ourselves, how highly we appreciate the practical bearing of this book; it is replete with precepts which ought to be treasured in the mind of the practitioner. The lectures are addressed to students, yet there is much instruction even for the man in extensive practice. For the reader, perhaps a more methodical arrangement of subjects would be preferable. In our analysis, therefore, we have taken the liberty occasionally of leaping onwards to a resumption of the subject under our review, that we might concentrate the points. We trust Dr. Todd will still proceed in these intricate investigations, as we are certain that they are the only legitimate mode of elucidating both the nature of disease, and the mutual influence of mind and organism, as far as the Creator has ordained that these mysteries *shall be* unveiled.

---

